# Microenvironment-Induced Non-sporadic Expression of the AXL and cKIT Receptors Are Related to Epithelial Plasticity and Drug Resistance

**DOI:** 10.3389/fcell.2018.00041

**Published:** 2018-04-17

**Authors:** Tiina A. Jokela, Agnete S. T. Engelsen, Agata Rybicka, Fanny A. Pelissier Vatter, James C. Garbe, Masaru Miyano, Crina Tiron, Dan Ferariu, Lars A. Akslen, Martha R. Stampfer, James B. Lorens, Mark A. LaBarge

**Affiliations:** ^1^Department of Biomedicine, University of Bergen, Bergen, Norway; ^2^Department of Population Sciences, Center for Cancer and Aging, City of Hope, Duarte, CA, United States; ^3^Centre for Cancer Biomarkers, University of Bergen, Bergen, Norway; ^4^Biological Systems and Engineering Division, Lawrence Berkeley National Laboratory, Berkeley, CA, United States; ^5^Regional Institute of Oncology, Iasi, Romania

**Keywords:** breast cancer, MEMA, microenvironment, epithelial plasticity, AXL, cKIT, drug resistance

## Abstract

The existence of rare cancer cells that sporadically acquire drug-tolerance through epigenetic mechanisms is proposed as one mechanism that drives cancer therapy failure. Here we provide evidence that specific microenvironments impose non-sporadic expression of proteins related to epithelial plasticity and drug resistance. Microarrays of robotically printed combinatorial microenvironments of known composition were used to make cell-based functional associations between microenvironments, which were design-inspired by normal and tumor-burdened breast tissues, and cell phenotypes. We hypothesized that specific combinations of microenvironment constituents non-sporadically impose the induction of the AXL and cKIT receptor tyrosine kinase proteins, which are known to be involved in epithelial plasticity and drug-tolerance, in an isogenic human mammary epithelial cell (HMEC) malignant progression series. Dimension reduction analysis reveals type I collagen as a dominant feature, inducing expression of both markers in pre-stasis finite lifespan HMECs, and transformed non-malignant and malignant immortal cell lines. Basement membrane-associated matrix proteins, laminin-111 and type IV collagen, suppress AXL and cKIT expression in pre-stasis and non-malignant cells. However, AXL and cKIT are not suppressed by laminin-111 in malignant cells. General linear models identified key factors, osteopontin, IL-8, and type VIα3 collagen, which significantly upregulated AXL and cKIT, as well as a plasticity-related gene expression program that is often observed in stem cells and in epithelial-to-mesenchymal-transition. These factors are co-located with AXL-expressing cells *in situ* in normal and breast cancer tissues, and associated with resistance to paclitaxel. A greater diversity of microenvironments induced AXL and cKIT expression consistent with plasticity and drug-tolerant phenotypes in tumorigenic cells compared to normal or immortal cells, suggesting a reduced perception of microenvironment specificity in malignant cells. Microenvironment-imposed reprogramming could explain why resistant cells are seemingly persistent and rapidly adaptable to multiple classes of drugs. These results support the notion that specific microenvironments drive drug-tolerant cellular phenotypes and suggest a novel interventional avenue for preventing acquired therapy resistance.

## Introduction

The confounding reality for anti-cancer treatments is the heterogeneity of tumors. Generated by genetic and adaptive epigenetic alterations in gene expression, tumor heterogeneity supports acquired resistance to anti-cancer treatments. Sporadic drug-tolerant states within subpopulations of cancer cells are rapidly achieved by activating drug-resistance genes, that are also implicated as stem cell-related genes, through chromatin modifications or transcriptional upregulation (Sharma et al., 2010; Shaffer et al., 2017). These epigenetic mechanisms provide rapidly acquired resistance and tumor cell persistence during treatment. Heterogeneity within the tumor microenvironment is a source of adaptive drug resistance that supports stem cell-like phenotypic plasticity in the tumor cells (Bissell and Labarge, 2005; LaBarge, 2010). However, the nature of these plasticity-inductive microenvironments remains elusive.

In normal tissues, stem cell-states are maintained in specialized microenvironments termed, niches. Epigenetic plasticity gene programs that are characteristic of regenerative stem cells responsive to tissue damage and inflammation are prominent in aggressive cancers with poor clinical outcome. These plasticity gene programs are triggered both by protective anti-tumor immune surveillance and inflammation, and the constant nutrient and oxygen deprivation characteristic of the chaotic tumor microenvironment that follows the breakdown of normal tissue architecture (Nieto, 2013). Tumor cells exploit these acquired stem cell traits to promote survival and enable flexibility to transition between different functional states such as epithelial-to-mesenchymal transition (EMT) (Bissell and Labarge, 2005; Thiery et al., 2009; LaBarge, 2010; Mora-Blanco et al., 2013). The connection between EMT and stem cell traits has been well studied in the epithelial cells of the mammary gland, an organ formed by branching morphogenesis, where epithelial plasticity is essential and where substantial cellular dynamics continue throughout adulthood (Petersen and Polyak, 2010). Regulators of EMT induce epithelial plasticity during mammary gland development and cancer progression (Mani et al., 2008; Guo et al., 2012). The importance of epithelial plasticity during the development of malignant breast cancer is evidenced by EMT gene signatures, which correlate with drug resistance, stem cell-like traits, basal breast cancer subtypes, metastasis and poor patient survival (Blick et al., 2010). The receptor tyrosine kinase (RTK), AXL, is a key driver of tumor cell EMT and is widely implicated in acquired drug-resistance to multiple cancer drug classes (Davidsen et al., 2017; Ferreira et al., 2017). Blockade of AXL inhibits the EMT program and reverses acquired drug resistance and metastasis (Gjerdrum et al., 2010; Kirane et al., 2015). AXL is an important therapeutic target currently being investigated in several cancer clinical trials (Antony and Huang, 2017). The RTK cKIT is enriched on mammary epithelial progenitor cells and increased expression was observed in high-risk breast tissue (Lim et al., 2009; Garbe et al., 2012). cKIT pathway activation is a driver in several cancers and it is related to acquired drug resistance (Javidi-Sharifi et al., 2015; Zhang et al., 2015; Lai et al., 2016; Pu et al., 2017). The ability of cells to modulate proteins related to stem cell-states such as these RTKs, is an example of epithelial plasticity, which can be useful for maintaining healthy tissue architecture in the normal context. Conversely, that same property is dangerous when coopted by cancer cells, as it promotes their survival and spread.

We hypothesized that sporadic stem cell-like states, which may be drug tolerant, are favored by specific microenvironment contexts. To address this, we functionally interrogated an isogenic human mammary epithelial cell (HMEC) progression series comprising primary normal (184, pre-stasis), immortal (184A1, non-malignant), and adenocarcinoma-forming (184AA3, tumorigenic) cells on combinatorial MicroEnvironment MicroArrays (MEMA) for induction of cKIT and AXL. The MEMA consisted of 228 distinct microenvironment features comprising different combinations of ECM, growth factors and cytokines. Hierarchical clustering, general linear modeling (GLM), and dimension reduction analyses were applied to identify plasticity-inductive microenvironments. Specific combinatorial microenvironments were shown to induce or maintain cKIT and AXL, activate an EMT-related gene expression program, and induce paclitaxel tolerance. The microenvironment components that were functionally predicted to induce AXL expression on MEMA, were found co-expressed by cells in breast tumor microenvironments adjacent to cells expressing AXL. We report evidence that sporadic drug-tolerance can result from phenotypic plasticity of carcinoma cells in response to different microenvironments.

## Results

### The normal and neoplastic mammary microenvironment

**Figure 1:**
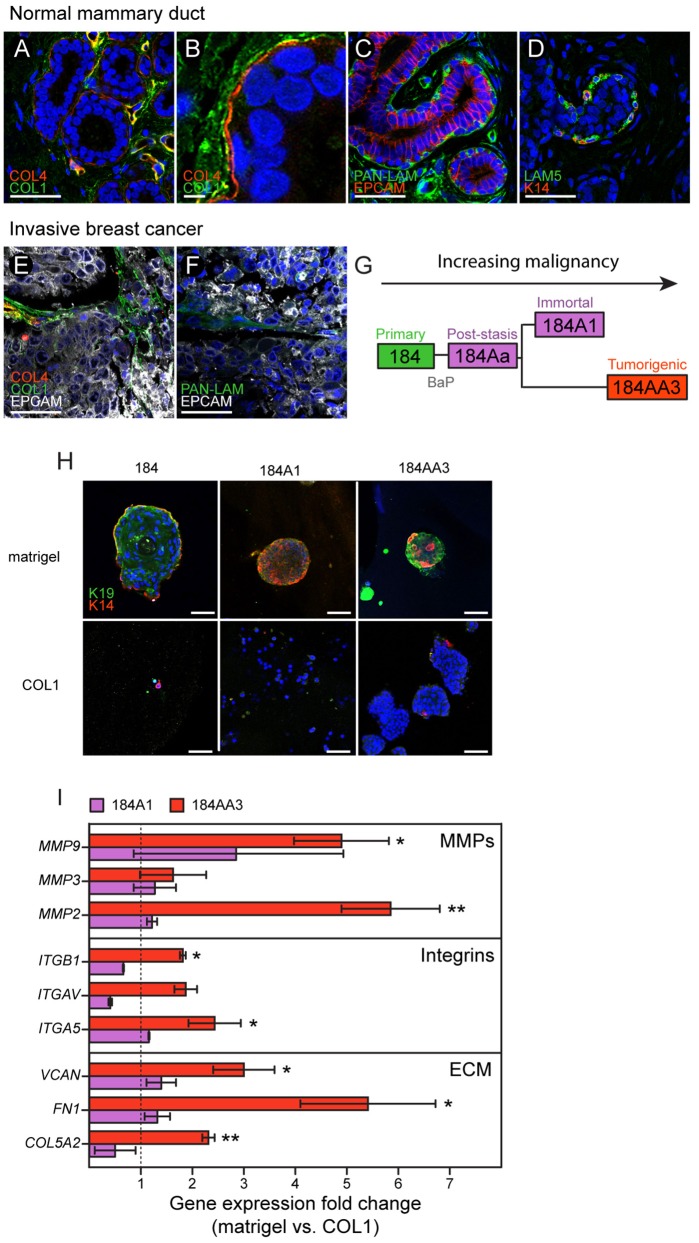
Human mammary epithelial cells from different stages in a malignant progression series exhibit unique growth characteristics in normal- and tumor-like microenvironments. Immunofluorescence staining of **(A–D)** normal and **(E,F)** invasive breast cancer tissue sections. ECM components; **(A,B,E)** COL4 (red), COL1 (green), **(C,F)** pan-laminin (LAM, green) and **(D)** LAM5 (green) in **(A–D)** normal mammary gland tissue and **(E,F)** invasive breast cancer, were stained with **(A–F)** nuclei marker Hoechst (blue), **(C,E,F)** epithelial cell marker (EPCAM) or with **(D)** myoepithelial cell marker (K14, red). **(G)** Diagram of the 184-progression series derivation. (H) Single cell suspensions of 184, 184A1, and 184AA3 cells were embedded in matrigel- and COL1-3D gels, after 12 days cells were fixed and stained with luminal cell marker (K19, green) and myoepithelial cell marker (K14, red). Images are representative of three individual experiments. **(A,C–F,H)** Bars represent 50 μm and **(B)** 5 μm. **(I)** Gene expression of microenvironment related genes (RT^2^Profiler™ PCR array, Human Epithelial to mesenchymal transition EMT, Qiagen) in 184A1 and 184AA3 cells cultured on matrigel (control = 1) or on COL1. Data represent mean ± SE, from two (184A1) or three (184AA3) individual experiments, statistical significance was calculated by using student *T*-test (^*^p < 0.05, ^**^p < 0.01).

Mammary epithelial ducts are encapsulated by a basement membrane that is enriched with laminins (LAM1 and LAM5) and type IV collagen (COL4). This matrix systematically regulates cell growth, induces lumen formation, and serves as a crucial polarity cue (Petersen et al., 1992). Examples of immunofluorescence staining in normal breast tissue demonstrate that epithelial cells are enveloped by basement membrane components COL4 (Figures 1A,B) pan laminin staining (PAN-LAM) and LAM5 (Figures 1C,D, respectively). Normal breast epithelia were clearly separated from type I collagen (COL1) that is most prevalent in the surrounding stroma (Figures 1A,B). The basement membrane is disrupted during breast cancer progression and local concentrations of COL4 and laminins decrease (Insua-Rodriguez and Oskarsson, 2016), while expression of matrix components characteristic of tissue remodeling increase (e.g., hyaluronan, HA, tenascin C, TNC, osteopontin, OPN, and fibronectin, FN1; Insua-Rodriguez and Oskarsson, 2016). COL1 accumulates and aligns at the epithelial-stromal borders of tumors (Provenzano et al., 2006). Immunofluorescence staining of invasive breast cancer tissues demonstrates that tumor cells are exposed to COL1 (Figure 1E), and only modest levels of PAN-LAM (Figure 1F). COL4 was absent from the tumor stroma, with only perivascular COL4 observed (Figure 1E). Accompanying these changes in ECM composition, heterogeneous breast cancer microenvironments are enriched with hormones (Garcia-Robles et al., 2013; Simões et al., 2015), growth factors (Mimeault et al., 2007; Ye et al., 2009; Zheng et al., 2014; Ho-Yen et al., 2015; Voudouri et al., 2015), cytokines (Esquivel-Velazquez et al., 2015; Weichhaus et al., 2015), chemokines (Palacios-Arreola et al., 2014) and cell adhesion proteins (Spivey et al., 2012; Beauchemin and Arabzadeh, 2013; Karousou et al., 2014; Yu and Elble, 2016).

### HMEC progression series for probing responses to normal- and stromal-like microenvironments

The 184 HMEC progression series provides a model of cancer progression comprising normal, finite lifespan, pre-stasis cells and derivative cell lines that range from non-malignant immortal non-malignant to malignant immortal cells (Figure 1G; Stampfer et al., 2013). The pre-stasis HMEC 184 strain was derived from normal reduction mammoplasty tissue of a 21-year old female with no pathological changes. Pre-stasis HMEC strains grown as described are known to possess luminal and myoepithelial cells and cells with progenitor activity (Garbe et al., 2009, 2012; Labarge et al., 2013). Finite post-stasis 184Aa were derived following benzo-a-pyrene (BaP) exposure of pre-stasis 184, and lack expression of the CKI p16^INK4a^ (Stampfer and Bartley, 1985; Brenner et al., 1998). The non-malignant immortal non-malignant cell line 184A1, which is wild-type for p53 and retinoblastoma (RB) protein, emerged from 184Aa as it approached replicative senescence, and exhibits a low level of genomic instability (Stampfer and Bartley, 1985; Walen and Stampfer, 1989). The tumorigenic cell line 184AA3 emerged from 184Aa following insertional mutagenesis that inactivated p53 function (Stampfer et al., 2003). It exhibits increased genomic instability and forms clinically relevant ER+ luminal adenocarcinomas in the mouse xenograft model (Stampfer et al., 2003; Hines et al., 2016). To evaluate how the HMEC progression series responds to normal-like and stroma-like microenvironments, we cultured single cell suspensions in laminin-rich ECM [lrECM (matrigel)] and COL1 3D gels, respectively. Normal 184 cells enriched for cKIT expression gave rise to growth arrested acini that have a lumen, with (K)eratin 14+ myoepithelial cells that are basal relative to K19+ luminal cells (Figure 1H), whereas growth in COL1 was negligible (Figure 1H). 184A1 and 184AA3 form solid, multi-lineage spheres in lrECM (Figure 1H). 184A1 exhibits modest growth in COL1 gels resulting in small colonies. In contrast, 184AA3-derived spheroids were large and proliferative in COL1 gels (Figure 1H). Gene expression analysis after 24 h growth on COL1 gels showed that tumorigenic 184AA3 cells, as compared to 184A1, upregulated expression of matrix metalloproteinases (*MMP2, MMP3*, and *MMP9*), integrins (*ITGB1, ITGAV*, and *ITGA5*) and matrix components [Versican (*VCAN*), *FN1* and type Vα2 collagen (*COL5A2*)] (Figure 1I), indicative of enhanced microenvironment-adaptive activity in the malignant cells.

### The relationship between cancer progression stage and plasticity marker expression in combinatorial microenvironment contexts

**Figure 2:**
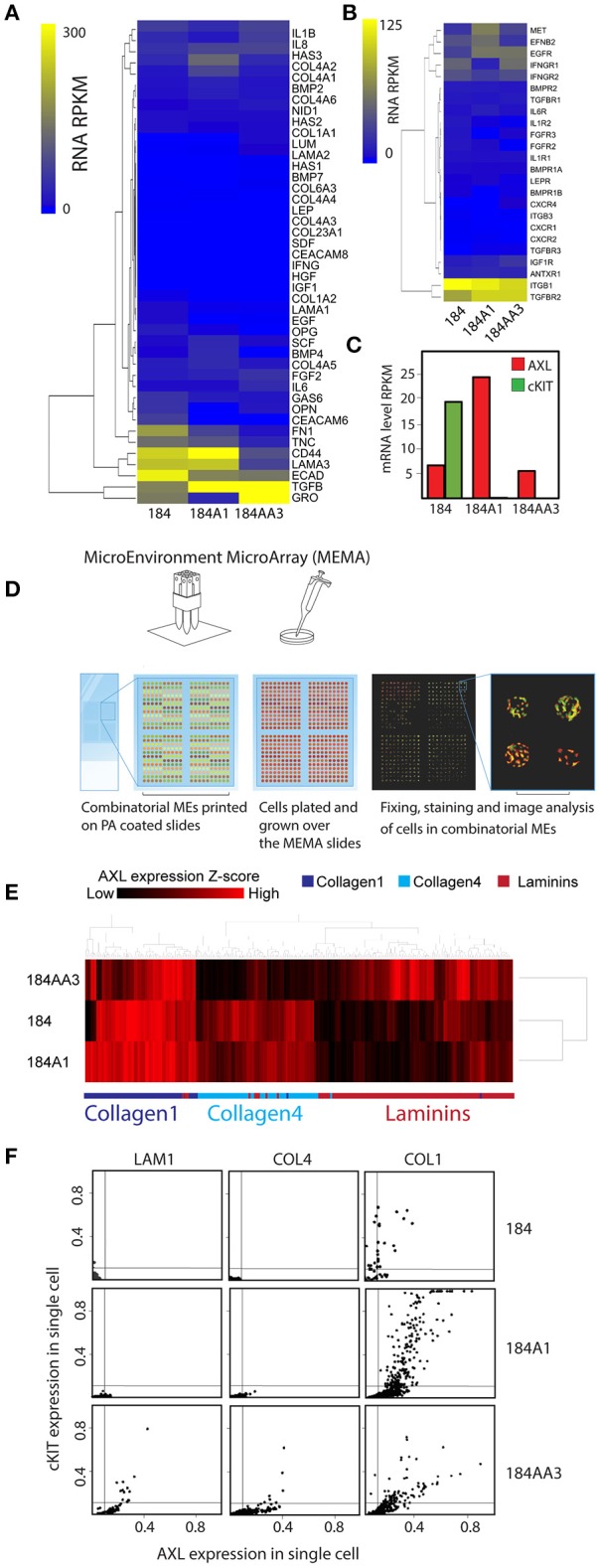
Non-sporadic induction of AXL and cKIT expression by combinatorial microenvironments. **(A,B)** Unsupervised hierarchical clustering of mRNA expression levels of genes in the 184 progression series corresponding the gene products that were printed on MEMA: **(A)** microenvironment proteins and **(B)** their known receptors. **(C)** mRNA expression level of *AXL* and *cKIT* in the184 progression. **(D)** Diagram of the MicroEnvironment MicroArray (MEMA) experimental design. MEMAs are printed on microscope slides coated with polyacrylamide (PA) gel. 228 unique extracellular microenvironments with 5–20 replicate spots are printed on one slide. Cells are cultured on the arrays and grown 48 h before fixing. AXL and cKIT are stained for immunofluorescence imaging, and image analysis is used to obtain single cell expression data in discrete microenvironment contexts. **(E)** Unsupervised hierarchical clustering of AXL expression z-scores as a function of microenvironment in the 184 progression series. Non-sporadic clustering of AXL expression by major ECM component of microenvironment was detected. **(F)** Scatter plot representation of AXL and cKIT expression in single cells, as a function of ECM components (LAM1, COL4, and COL1) in the 184 progression series.

We next asked whether induction of phenotypes associated with plasticity and drug-tolerant states is sporadic (equally likely to occur in all microenvironment contexts), or whether those states are associated with specific microenvironments (microenvironment-induced). MEMA were used previously to identify combinatorial microenvironments that induce and maintain stem- and differentiated-states in HMEC (LaBarge et al., 2009), and microenvironments that modulate lapatinib activity in HER2-amplified breast, lung, and prostate cancer (Lin et al., 2017). We applied this principle to determine whether cKIT and AXL were expressed in a microenvironment dependent manner. Individual microenvironment components were selected based on their enrichment in normal and cancer microenvironments. In order to recapitulate simplified normal- or tumor-like microenvironments purified COL1, Laminin-111 (LAM1), COL4, and LAM1+laminin-332 (LAM5) were mixed pairwise with OPN, HA, TNC, FN1, bone morphogenetic protein−2/7 (BMP-2/7), BMP-4, carcinoembryonic antigen related cell adhesion molecule 6 (CEACAM6), CEACAM8, CD44, type XXIIIα1 collagen (COL23A1), E-cadherin (ECAD), epidermal growth factor (EGF), fibroblast growth factor 2 (FGF-2), growth arrest-specific 6 (GAS-6), hepatocyte growth factor (HGF), insulin-like growth factor (IGF1), interferon-γ (IFN-γ), interleukin-1β (IL-1β), IL-6, IL-8, leptin, melanoma growth stimulating activity alpha (GRO1), nidogen1, lumican, osteoprotegerin (OPG), stem cell factor (SCF), stromal derived factor-1β (SDF-1β), and tumor growth factor β (TGFβ) to make a total of 228 unique combinations that were printed in 10-fold replicate features. RNA sequencing of the 184 progression series was performed to characterize the baseline levels of expression of the genes corresponding to the proteins printed on MEMA (Figure 2A) and to their known receptors (Figure 2B). Significant differences were not detected in expression of any of these genes. However, levels of *AXL* gene expression are 5 fold higher in 184A1 cells compared to the other cells and *cKIT* gene expression was detected only 184 cells (Figure 2C).

**Figure 3:**
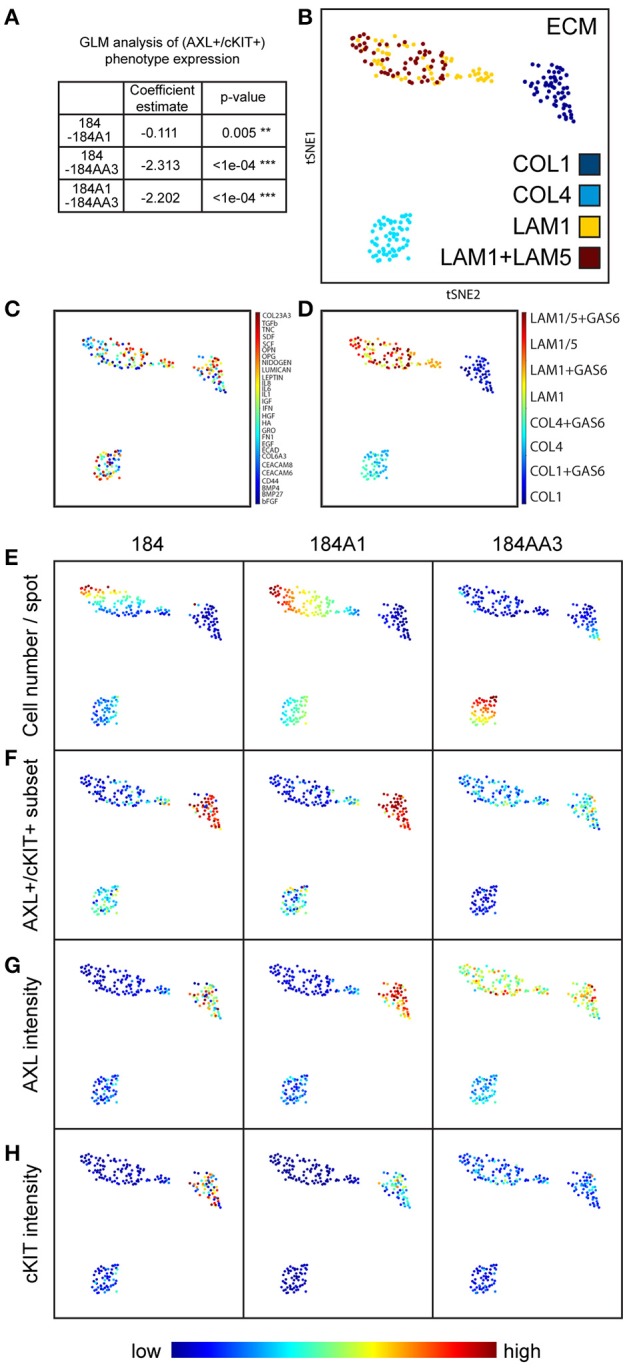
Visualizing the impact of microenvironment on higher-order cell phenotypes, including AXL and cKIT expression. **(A)** Table representing GLM analysis of expression of the AXL+/cKIT+ phenotype in different microenvironments and significant differences was detected in patterns of microenvironment-phenotype associations between comparisons of all progression stages. ^**^p ≤ 0.01, ^***^p ≤ 0.001. **(B–H)** Dimension reduction and visualization of microenvironment-driven phenotypes with tSNE visualization. Each point represents a unique combinatorial microenvironment, and the distance between any two points reflects similarity of the cellular phenotype that is begotten by the microenvironment. The characteristics that were measured in cells to establish phenotype were: % of cells that are AXL^+^/cKIT^+^, mean AXL, and cKIT fluorescent intensity in subpopulation and in ungated population, cell count/spot, -eccentricity, -solidity. **(B–D)** Shows the composition of each microenvironment, where **(B)** shows distribution of the major ECMs, **(C)** shows the distribution of the soluble factors, and **(D)** shows the distribution of GAS6 among the major ECM. The major ECM is a key driver of microenvironment-imposed phenotypes. Even GAS6, the cognate ligand of AXL does itself impact the tSNE distributions. **(E–H)** Show how specific aspects of cell phenotype distribute with microenvionment: **(E)** cell number per spot, **(F)** AXL^+^/cKIT^+^-subpopulation size, **(G)** mean AXL intensity, and **(H)** mean cKIT intensity.

Cells have a dynamic and reciprocal relationship with their microenvironment, and one would expect cells to gradually modify their microenvironment following initial exposure. Thus, in order to measure the impact of the printed combinatorial microenvironments on the 184 HMEC progression series, they were cultured on MEMA for only 48 h. After fixing and staining for AXL and cKIT protein expression, every MEMA feature was imaged, and single cell data were obtained through use of marker-based watershed segmentation (Figure 2D). In addition to protein levels, cell-segmentation enabled assessment of eight morphological properties of cells that were evaluated independently. Unsupervised clustering of AXL expression Z-scores as a function of microenvironment revealed that AXL expression in 184 and 184A1 was mainly observed in COL1-rich microenvironments, with less expression in COL4-rich ones (Figure 2E). In contrast, AXL expression was high in COL1- and LAM1-rich microenvironments in malignant 184AA3 cells (Figure 2E). We show an example of AXL and cKIT expression in cells of the progression series, at the single cell level, on three single component microenvironments: COL1, COL4, and LAM 1 (Figure 2F). These data show that microenvironments that impose expression of the RTKs may not do so uniformly, that it is more a case of triggering a percentage of the cells to express those proteins instead of shifting the mean of the population, which underscores the importance of single cell analysis. Single cell data also revealed that AXL and cKIT expression in malignant 184AA3 cells was overall more heterogeneous than in 184 and 184A1 cells (Figure 2F). General linearized modeling (GLM) confirmed that the coefficient of variance describing the percentage of AXL and cKIT-expressing cells in a given microenvironment between 184 and 184A1 cells was low, while the variance between 184 and 184AA3, and 184A1 and 184AA3 was more than two-magnitudes greater (Figure 3A). Thus, whereas expression of these RTKs in normal and non-malignant HMEC is tightly regulated by microenvironment, the malignant cells are effected but not fully restricted by microenvironment.

Next, we applied tSNE to project all the dimensions in 2D and visualize the data, a method particularly sensitive to the types of non-linear relationships that are common in biological data to visualize the relationship between microenvironment and RTK expression (Amir et al., 2013). Microenvironments were readily clustered by the primary ECM component (Figure 3B). Whereas, there was no clear clustering driven by presence of soluble ligands (Figure 3C), nor by GAS6 (Figure 3D), which is the cognate ligand for AXL. After only 4 h, array features were fairly uniformly bound (with a potentially universal attachment preference for COL1) (data not shown), but differences in cell number per feature changed with time, revealing some matrix-type preferences that were progression stage dependent by 48 h. Normal and non-malignant HMEC preferentially increased in cell number/spot on LAM1 and COL4, and malignant cells on COL4 (Figure 3E). The majority of AXL and cKIT expression in normal and non-malignant cells was detected in cells cultured on COL1-containing microenvironments, with some weak enrichment also seen on COL4-containing (Figures 3F–H). In comparison, malignant cells expressed high amounts of AXL and cKIT, in COL1- and LAM1-containing microenvironments, but COL4 was the only ECM that was not associated with expression of those RTKs (Figures 3F–H). 184AA3 was more likely to have significantly greater proportions of AXL and cKIT expressing cells in microenvironments that included COL1 or LAM1 (Figures 3F–H), and GLM analysis showed significantly more variance (expression of AXL+/cKIT+ population) in COL1 and LAM1 including microenvironments compared to other ECMs (Table 1). Collectively this cell-based functional analysis of microenvironment-phenotype associations showed that COL1-rich stroma-like and LAM1-rich normal-like microenvironments enabled the induction of AXL and cKIT-expression in malignant cells, whereas their expression in normal and non-malignant cells was far more restricted. These data provide a functional rationale for normal epithelial cell segregation from the stromal microenvironment by the basement membrane, and reveal an inherent plasticity of epithelia that engages components of stem cell-related signaling pathways when exposed to stromal ECM, for example during trauma or disease. Malignant cells, by comparison, readily switch between stem- and resistance-related pathways in microenvironments that normally suppress plasticity, e.g., LAM1-rich contexts.

**Table 1:** Effect of ECM on expression of AXL^+^/cKIT^+^-phenotype in 184AA3.

**ECM**	**Co-efficient estimate from GLM analysis**	***P*-value**
COL1-COL4	−1.64569	<0.001^***^
COL1-LAM1	0.04385	0.0621
COL1-LAM1/LAM5	−0.37975	<0.001^***^
COL4-LAM1	1.53714	<0.001^***^
COL4-LAM1/LAM5	1.26594	<0.001^***^
LAM1-LAM1/LAM5	−0.2712	<0.001^***^

*GLM analysis with Tuckey's post-hoc test*.

*** *p ≤ 0.001*.

### OPN, IL-8, and COL6A3 promote states consistent with drug-tolerance in malignant cells

**Figure 4:**
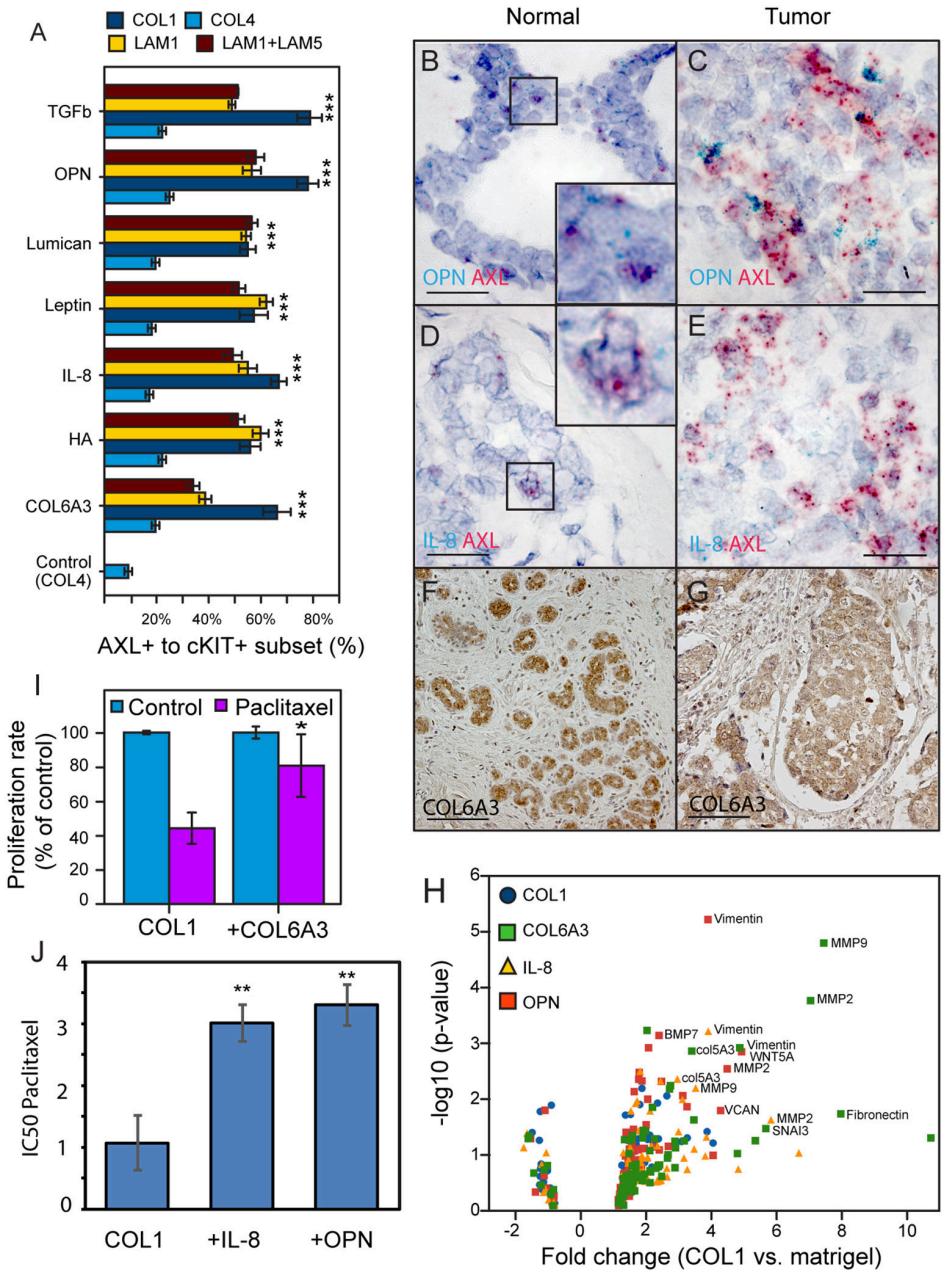
Identification and *in vivo* validation of microenvironment factors that impose AXL and cKIT expression phenotypes in malignant breast cancer cells. **(A)** AXL^+^/cKIT^+^-184AA3 phenotype expression in MEMA experiments was analyzed by GLM, and the most significant (Tuckey's *post-hoc* test, *p* < 2e-16^***^) microenvironment supplemental factors combined with different ECM are presented in bar graph format. **(B,C)** Co-expression of *OPN* (cyan), and *AXL* (red) were determined by RNA *in situ* hybridization of normal human mammary gland tissue **(B)**, and TNBC **(C)** specimens. Co-expression *IL-8* (cyan), and *AXL* (red) in normal human mammary gland tissue **(D)**, and TNBC (E) specimens. Scale bar represent 20 μm **(B–E)**. Expression of COL6A3 in normal human mammary gland tissue **(F)**, and TNBC **(G)** specimens were assayed by IHC-P. **(F,G)** Scale bar = 100 μm. Counterstaining by hematoxylin **(B–G)**. **(H)** Volcano plot represent EMT related gene expression (RT^2^Profiler™ PCR array, Human Epithelial to mesenchymal transition EMT, Qiagen) in 184AA3 cell cultured (24 h) on COL1, with or without OPN, IL-8, or COL6A3 was compared to expression profile of 184AA3 cells on matrigel. Results represent mean of three individual experiments, and *p*-values are calculated by comparing each gene expression in each group with the matrigel group, ^***^≤0.001. **(I)** To study drug resistance, 184AA3 cells were cultured on COL1 coated dishes supplemented with or without COL6A3, and treated with 0.1 μM paclitaxel. Data represent EdU positive cells as a percentage of total cells compared to COL1 control culture. Results represent mean ± SD in 6 individual experiments, significance between COL1 and COL1+COL6A3, ^*^p = 0.02. **(J)** To study impact of OPN and IL-8 on paclitaxel IC50 values (μg/ml), 184AA3 cells were cultured on COL1 with or without OPN or IL-8. Cells were treated with Paclitaxel (ranging from 0.001 to 1 μg/ml). Results represent mean ± SD in 3 individual experiments, significance between IC50 values, ^**^p < 0.01.

GLM analysis of the 184AA3 MEMA showed that TGFβ, OPN, lumican, leptin, IL-8, HA and COL6A3 were significantly associated with increased frequency of the AXL and cKIT phenotype (Figure 4A). To further investigate this differential plasticity-inductive effect, we focused on OPN, IL-8, and COL6A3, which were associated with the largest proportion of AXL-expressing 184AA3 cells. We examined the expression of these plasticity-inductive factors in normal human mammary gland and in triple negative breast cancer (TNBC) tissue sections (Figures 4B–G). By RNA *in situ* hybridization the expression of *OPN* and *IL-8* were found to co-localize with the rare population of cells with high *AXL* expression in normal tissue (Figures 4B,D) and, as well, *OPN* and *IL-8* expressing cells were also found in the vicinity of the *AXL* positive tumor cells in the TNBC tissues (Figures 4C,E). Expression of COL6A3 was prominent in the normal epithelia (Figure 4F), and also in the malignant epithelium of TNBC (Figure 4G). This result contrast with the detection of mature type VI collagen (COL6) fibrils, which were detected strictly in the stromal compartments in normal mammary tissues as well as in TNBC specimens (data not shown), and might indicate a particular role of the α3 chain of COL6 in homeostasis of mammary epithelia. Thus, *in vivo* cells expressing *OPN, IL-8* and COL6A3 are co-located with cells expressing AXL in normal and tumor contexts.

AXL is associated with drug resistance and metastatic spread of breast cancer (Li et al., 2015; Antony and Huang, 2017; Davidsen et al., 2017). We evaluated expression of a panel of EMT- and stem cell-related genes (Table 2) in 184AA3 cells cultured on lrECM (matrigel), COL1, or COL1 supplemented with OPN, IL-8 or COL6A3. A number of EMT related genes were upregulated by the COL1 microenvironment, and the upregulation was enhanced when COL1 was supplemented with any of the three factors (Figure 4H). These results indicate that OPN, IL-8, and COL6A3 in COL1- and LAM1-rich contexts non-sporadically induce AXL and cKIT expression, and gene expression consistent with engagement of EMT-related programs in the COL1-rich context, in tumorigenic HMEC, which may induce drug-tolerant states. To determine if plasticity-inductive microenvironments were sufficient to increase drug-tolerance, 184AA3 cells were cultured on COL1-only, COL1+COL6A3, COL1+IL-8, or COL1+OPN microenvironments and treated with paclitaxel. These microenvironments significantly increased tolerance to the drug, consistent with the notion that epithelial plasticity enables non-sporadic induction of drug-tolerant states (Figures 4I,J).

**Table 2:** EMT related gene expression profile (RT^2^Profiler™ PCR array, Human Epithelial to mesenchymal transition EMT, Qiagen) of 184AA3 cell cultured (24 h).

**Gene symbol**	**COL1**	**+OPN**	**+IL-8**	**+COL6A3**
CAMK2N1	0.59	0.59	0.53	0.55
RGS2	0.68	0.57	0.71	0.56
MAP1B	0.68	0.63	0.74	1.53
GEMIN2	0.69	0.94	1.00	0.90
FGFBP1	0.69	0.79	0.77	1.18
TGFB2	0.69	0.76	0.51	1.05
ZEB2	0.70	0.96	1.38	1.45
ESR1	0.72	1.36	1.91	2.28
STEAP1	0.74	1.39	1.55	1.57
NUDT13	0.75	0.95	1.03	0.61
SNAI2	0.82	3.99	1.08	4.76
PTP4A1	0.82	1.06	0.88	0.81
EGFR	0.88	0.97	1.08	1.07
OCLN	0.88	1.13	0.91	1.37
SMAD2	0.89	1.09	1.23	1.03
RAC1	0.89	1.04	1.06	1.12
DSP	0.91	1.34	1.32	1.29
DESI1	0.91	0.98	1.01	0.97
KRT19	0.93	1.01	1.40	1.87
SPP1	0.93	1.08	0.84	0.70
SNAI1	0.93	2.31	1.47	1.27
PTK2	0.94	1.12	1.21	1.20
GSK3B	0.95	1.02	1.13	1.35
KRT7	0.95	0.77	0.79	0.75
NODAL	0.97	1.37	2.54	1.41
TGFB3	0.98	1.19	1.10	0.90
VPS13A	0.98	1.36	1.61	1.29
IL1RN	1.00	1.35	0.97	2.15
TIMP1	1.00	1.26	1.13	1.25
CAV2	1.00	1.08	1.16	1.16
TSPAN13	1.02	1.16	1.31	1.16
TMEFF1	1.02	1.15	1.30	1.78
TCF3	1.05	1.21	1.00	1.05
BMP7	1.11	2.29	1.73	1.40
FZD7	1.12	1.34	1.30	1.48
STAT3	1.12	1.26	1.10	1.20
TWIST1	1.14	1.28	1.12	1.15
CALD1	1.15	1.49	1.65	1.45
ERBB3	1.21	1.20	1.36	1.45
AHNAK	1.22	1.32	1.47	1.45
AKT1	1.23	1.06	1.17	0.96
F11R	1.24	1.19	1.22	1.19
DSC2	1.25	1.73	1.93	2.20
CTNNB1	1.25	1.40	1.38	1.41
NOTCH1	1.27	1.56	1.33	1.17
TCF4	1.28	1.57	2.90	2.08
ILK	1.29	1.24	1.62	1.57
ZEB1	1.34	1.36	1.91	2.56
CDH1	1.34	1.30	1.55	2.01
ITGB1	1.36	1.94	2.00	2.65
PLEK2	1.36	1.45	1.60	1.91
MSN	1.39	1.66	1.69	1.79
ITGAV	1.41	1.67	1.78	3.39
COL1A2	1.43	1.89	3.17	2.57
FOXC2	1.43	1.52	1.33	1.31
TFPI2	1.43	1.14	1.49	1.60
BMP2	1.45	1.11	1.85	2.37
MST1R	1.48	1.41	1.79	1.40
JAG1	1.57	1.65	2.24	2.81
BMP1	1.62	1.68	2.05	2.00
SERPINE1	1.64	1.47	2.23	2.01
TGFB1	1.69	1.52	1.76	1.57
IGFBP4	1.75	1.33	1.75	1.12
MMP3	1.79	1.74	3.39	3.08
KRT14	1.84	3.18	3.03	1.82
TMEM132A	1.93	1.26	1.41	2.03
COL5A2	2.00	2.38	2.87	3.32
SNAI3	2.03	2.59	3.78	5.66
VCAN	2.07	4.23	3.75	5.33
ITGA5	2.12	1.76	2.54	2.79
GNG11	2.24	1.97	2.33	2.62
SPARC	2.28	1.84	3.07	2.80
VIM	2.53	3.84	3.83	4.84
FN1	3.00	2.12	4.79	8.02
WNT5A	3.18	4.90	6.69	10.86
MMP9	3.80	3.04	3.44	7.49
MMP2	4.00	4.44	5.81	7.06

*Fold expression presented in table were calculated using the formula 2^(−ΔΔCt)^, where ΔΔC_t_ is ΔC_t(sample)_−ΔC_t(matrigel sample)_, ΔC_t_ is C_t(gene of interest)_−C_t(average from control gene setup)_, and C_t_ is the cycle at which the detection threshold is crossed*.

## Discussion

Here we provide evidence in a breast cancer progression series, that specific tumor-associated microenvironments favor induction of two RTKs implicated in plasticity and drug-tolerant states, in a non-sporadic manner. COL1 is found extensively in tumor stroma, and when combined with certain other common tumor microenvironment proteins (i.e., COL6A3, OPN, and IL-8) the frequency of AXL-expressing cells significantly increased. We showed this functionally on MEMA, in follow up validation cell culture experiments, and using RNA *in situ* hybridization and immunohistochemistry, we demonstrated coordinated expression of these microenvironment factors with AXL expressing cells in breast tumors. Microenvironment proteins such as ECM molecules are not directly targeted by currently approved anti-cancer therapeutics, and they have long *in vivo* half-lives, e.g., the half-life of COL1 is estimated between 14 and 400 years depending on the tissue (Verzijl et al., 2000). Thus, microenvironment-imposed reprogramming could explain why resistant cells are seemingly persistent and rapidly adaptable to multiple drugs. Drug-induced sporadic transcription of a number of other gene markers that are implicated in drug-tolerance was shown, *AXL* among them, though they did not account for microenvironment context (Shaffer et al., 2017). We reported previously that the response of HER2-amplified cells to the HER2-targeted drug lapatinib is partly determined by combinatorial microenvironments (Lin et al., 2017). We speculate that microenvironment-induced drug-tolerance via induction of plasticity-related genes and proteins is a widespread mechanism. Because specific microenvironments are associated to enable certain cellular phenotypes (e.g., AXL and cKIT states) the mechanism is not sporadic, and it suggests an avenue forward for circumventing drug-tolerance.

AXL also is implicated as having a functional role in cells that possess cancer stem cell (CSC) activity. AXL expression is a strong negative prognostic factor for human breast cancer survival and its expression is associated with spread of metastatic breast cancer (Gjerdrum et al., 2010). AXL expression is upregulated during EMT, and the EMT gene program is associated with cells that exhibit normal stem cell and cancer stem cell (CSC) activity (Liu and Fan, 2015). It enhances migratory activity of pre-malignant breast epithelial cells and contributes to breast cancer cell extravasation into lungs (Vuoriluoto et al., 2011). cKIT also is associated with cells that possess mammary progenitor activity (Lim et al., 2009; Garbe et al., 2012), so it is not surprising that many of the microenvironments that induced AXL also induced cKIT, as well as the EMT gene signature. Thus, prior to this study epithelial plasticity, and the underlying EMT-related gene programs, have been widely discussed in the context of metastatic spread. The MEMA platform is probably not the correct model for understanding processes related to metastasis, but we demonstrate here its utility in examining the roles of microenvironment in drug-tolerance. MEMA have proven useful in functionally defining putative normal stem cell niche components using the juxtaposition of lineage specific keratins as cell fate markers (LaBarge et al., 2009). From these new results, we speculate that CSC niche components also could be functionally identified using the MEMA platform.

In the mature mammary gland, the basement membrane is located between the epithelium and stroma, compartmentalizing breast tissue components. COL4, LAM1, and LAM5 are important basement membrane constituents that promote attachment of epithelial cells and maintenance of epithelial polarity (Kleinman et al., 1981), while stroma is rich in COL1. The MEMA approach revealed that normal HMEC express AXL and cKIT in the COL1-rich microenvironments. This suggests that breakdown of normal tissue compartmentalization and exposure to COL1-rich stromal ECM is a plasticity signal for differentiated mammary epithelial cells. While stem cell traits are a common feature of malignant carcinoma, the reacquisition of stem cell properties by normal differentiated epithelial cells is poorly understood (Blanpain and Fuchs, 2014). Our results reveal that this may be regulated at the level of tissue organization through distinct combinatorial cell-cell and cell-ECM signals.

OPN, IL-8, and COL6A3 exposure upregulated EMT-related genes and COL6A3 supported breast cancer cell drug resistance. OPN is an N-linked glycoprotein and functions as an extracellular structural protein in many tissues. OPN expression is relatively low in normal mammary gland but it is induced during lactation and involution (Insua-Rodriguez and Oskarsson, 2016). OPN was reported to be part of the hematopoietic and neural stem cell niche (Haylock and Nilsson, 2006). IL-8 is a pro-inflammatory and pro-angiogenic factor, and is strongly associated with cancer progression. Genetic variation and increased expression of IL-8 correlates with increased risk of breast cancer as well as poor prognosis (Snoussi et al., 2006; Milovanovic et al., 2013). IL-8, among other cytokines, has been linked to regulation of the breast CSC state, and IL-8 can stimulate CSC self-renewal (Korkaya et al., 2011; Palacios-Arreola et al., 2014). COL6 is a widely distributed ECM macromolecule that plays a crucial role in tissue development, it was reported to be part of the normal breast and breast cancer microenvironments (Ferguson et al., 1992; Karousou et al., 2014). Upregulation of COL6 was shown to generate a microenvironment that promotes tumor progression (Chen D. et al., 2013). COL6 is a heterotrimer composed of three genetically distinct polypeptide chains: α1, α2, and α3 i.e., COL6A3. COL6A3 is the largest of these three chains and the cleavage of the C5 domain, also called endotrophin, has a crucial role in breast cancer development, and it is a ligand for ANTXR1-receptor, which is a putative biomarker for breast CSC (Chen P. et al., 2013; Karousou et al., 2014). COL6A3 represents a frequently mutated gene in triple negative breast cancers (Cancer Genome Atlas Network, 2012; Curtis et al., 2012; Shah et al., 2012). Based on our cell-based functional experiments, we suggest that OPN, IL-8 and COL6A3 are part of a CSC niche.

Cooption of epithelial plasticity mechanisms has emerged as a central challenge for current cancer treatments. In spite of advances in cancer therapies, most cancer patients still do not experience lasting clinical benefit. Tumor cells invariably elude treatment; reemerging as advanced, disseminated malignancy that is associated with increased mortality. This study highlights how malignant carcinoma cells adapt to different microenvironments by activating drug resistance via clinically actionable RTKs. Hence a deeper understanding the interplay between malignant cells and a dynamic microenvironment, and the underlying signaling pathways will inform new combination therapy approaches to prevent resistance.

## Materials and methods

### Contact for reagent and resource sharing

Further information and request for resources and reagents should be directed to and will be fulfilled by the lead contact, Jim Lorens (Jim.Lorens@uib.no) or Mark LaBarge (mlabarge@coh.org).

#### Cell culture

Human mammary epithelial cells were cultured in M87A media supplemented with cholera toxin at 0.5 ng/ml (Sigma-Aldrich) and oxytocin at 0.1 nM (Bachem, Switzerland) (Garbe et al., 2009). Cells were isolated from reduction mammoplasty specimen 184, a 21 year old Caucasian female, and maintained as previously described (Garbe et al., 2009; Labarge et al., 2013). Pre-stasis, passage 4,184 cells were sorted by flow cytometry, and a cKIT positive progenitor subpopulation was used for experiments. Immortal cell lineages were derived by using the chemical carcinogen benzo(a)pyrene (BaP) to overcome stasis. The BaP treated post-stasis 184Aa lineage clonally overcame the immortalization barrier to generate the non-malignant immortal 184A1 line (Stampfer and Bartley, 1985). The clonal tumorigenic cell line 184AA3 emerged from 184Aa following insertional mutagenesis in the p53 locus (Stampfer et al., 2003). Cells were cultured on 2D plastic dish (unless otherwise mentioned). In 3D cultures, a single cell suspension was embedded in growth factor reduced matrigel (Corning) or 1.5 mg/ml COL1 gel (rat tail, non pepsinized, 5 mg/ml, Ibidi), Culture medium was changed every other day, and after 12 days cells and gel were fixed and stained.

#### *In vivo* human tissue studies

The archival formalin fixed paraffin embedded (FFPE) tissues used in this study originates from the Department of Pathology, Haukeland University Hospital, Bergen, Norway, and the Regional Institute of Oncology, Iasi, Romania. Tissue histology and tumor classification were verified by trained pathologists at the respective institutions. Tissues from Haukeland University hospital has ethical approval REK (Regional Ethics Committee #2014/1984), and tissues from Regional Institute of oncology has approval from Ministerul sanatatii, IRO, Cod Fiscal 29067408.

#### Flow cytometry

One hundred and eighty four passage 4 HMECs were cultured close to confluence and trypsinized. After that fluorescein conjugated Anti-CD117/cKIT-antibody (Biolegend, clone 104D2, 1:50) was added to cells in media for 25 min−1 h on ice, cells were washed with cold PBS and sorted with FACS Vantage DIVA or FACS Aria SORP (Becton Dickinson).

#### MicroEnvironment MicroArray (MEMA)

MEMA method is comprehensively presented here (Lin et al., 2012; Lin and LaBarge, 2017). Briefly, polyacrylamide (PA) gels were made on standard glass microscope slide etched with 0.1M NaOH. Slides were covered with 3-Aminopropyltriethoxysilane (APES, Sigma-Aldrich), and after 5 min slides were soaked in distilled H_2_O. Then incubated 30 min at 0.5% Glutaraldehyde (Sigma-Aldrich) solution in PBS. After this, slides were dried and polyacrylamide gel 350 μl/slide was pipette on the slide and covered with cover glass. PA gel solution contained 5% acrylamide (Sigma-Aldrich) and 0.15% Bis-Acrylamide (VWR), final gel modulus was 4,470 ± 1,190 Pa. The MEMA master plate was prepared by diluting the ECM-combinations with printing buffer composed of 100 mM Tris-Acetate/20% glycerol/0.05% TritonX-100 pH5.2. Protein information and used concentrations used are shown in (Table 3). SpotBotIII microarrayer (ArrayIt, CA, USA) was used to perform printing, with 5–20 replicate spots of each microenvironment were printed. After printing MEMAs were placed into 4-well plates (Nunc) and first washed with PBS with 50 U/ml of penicillin and 50 U/ml streptomycin (Gibco), followed by a second wash with cell culture medium. Cells were diluted to the desired concentration into 5 ml of media, plated over the MEMA slide, and incubated at +37°C with 5% CO_2_. After 4 h one replicate MEMA was fixed with methanol:acetone (1:1) at −20°C for 20 min, to indicate cell attachment on MEMAs. For replicate MEMA, non-attached cells were washed away with culture media and fresh media was added to wells. After 24 h the media was changed again and after 48 h MEMAs slides were fixed, as described above. Fixed MEMA were blocked with PBS, 5% normal goat serum (Invitrogene), 0.1% Triton X-100 (Sigma-Aldrich), and incubated with anti-AXL (1:200, 10c9) and anti-cKIT (1:200, CD117, Biolegend) overnight at 4°C, then visualized with fluorescent secondary antibodies (VWR), and DRAQ5 DNA dye (Cell signaling). MEMA slides were imaged with InnoScan 1100 (Innopsys) or LSM710 confocal microscope (Carl Zeiss).

**Table 3:** Key resources table.

**Reagent of resource**	**Source**	**Identifier**	**Notes**
**ANTIBODIES**			
Anti-CD117 (cKIT)	Biolegend	313201	
Anti-AXL (10c9)	BerGenBio, Bergen, Norway	N/A	
Cytokeratin 14 antibody	Thermo scientific	PA5-16722	
Anti-Cytokeratin 19	Abcam	AB7754	
Anti-Collagen type IV	Merck millipore	MAB3326	
Anti-Collagen I	Abcam	AB34710	
Anti-Laminin-5	Merck millipore	MAB19562	
Anti-Laminin	Sigma-aldrich	L9393	
Anti-human CD326 (EPCAM)	Biolegend	34202	
Anti-AXL (mAb: 1H12)	BerGenBio, Bergen, Norway	N/A	
Anti-COL6A3	Novus biologicals	NBP-71566	
**BIOLOGICAL SAMPLES**			
The human FFPE-healthy mammary gland and breast cancer tissues	The Department of Pathology, Haukeland University Hospital, Bergen, Norway, and the University Hospital of Iasi, Iasi, Romania	N/A	
**Chemicals, peptides, and recombinant proteins**			**Concentration in MEMA, Reference; relevance in cancer**
Bone morphogenetic protein 2/7 heterodimer (BMP-2/7)	R&D systems	3229-BM/CF	1 μg/ml, (Ye et al., 2009)
Bone morphogenetic protein 4 (BMP-4)	R&D systems	113-BP/CF	1 μg/ml, (Ye et al., 2009)
Carcinoembryonic antigen-related cell adhesion molecule 6 (CEACAM6)	R&D systems	3934-CM-050	1 μg/ml, (Beauchemin and Arabzadeh, 2013)
Carcinoembryonic antigen-related cell adhesion molecule 8 (CEACAM8)	Abnova	H00001088-P01	1 μg/ml, (Beauchemin and Arabzadeh, 2013)
CD44	R&D systems	3660-cd	1 μg/ml, (Karousou et al., 2014)
Collagen I (COL1)	Sigma-Aldrich	C8919	100 μg/ml, (Insua-Rodriguez and Oskarsson, 2016)
Collagen IV (COL4)	Sigma-Aldrich	C5533	100 μg/ml, (Insua-Rodriguez and Oskarsson, 2016)
CollagenXXIIIA1 (COL23A1)	R&D systems	4165-CL	1 μg/ml, (Spivey et al., 2012)
CollagenVIα3 (COL6A3)	MyBiosource	MBS958856	1 μg/ml, (Karousou et al., 2014)
E-Cadherin (ECAD)	Sigma-Aldrich	E2278	1 μg/ml, (Yu and Elble, 2016)
Epidermal growth factor (EGF)	Sigma-Aldrich	E9644	1 μg/ml, (Voudouri et al., 2015)
Fibroblast growth factor basic (FGF-2)	R&D systems	233-FB-025	1 μg/ml, (Zheng et al., 2014)
Fibronectin (FN1)	Sigma-Aldrich	f2518	100 μg/ml, (Insua-Rodriguez and Oskarsson, 2016)
Growth arrest specific 6 (GAS-6)	R&D systems	885-GS-050	1 μg/ml, (Mc Cormack et al., 2008)
Hepatocyte growth factor (HGF)	R&D systems	294-HG-005	1 μg/ml, (Ho-Yen et al., 2015)
Hyaluronan HMW (HA)	R&D systems	GLR002	100 μg/ml, (Karousou et al., 2014)
Insulin like growth factor-1 (IGF1)	R&D systems	291-Gi-250	1 μg/ml, (Voudouri et al., 2015)
Interferon- γ (IFN-γ)	Gibco	PHC4031	1 μg/ml, (Esquivel-Velazquez et al., 2015)
Interleucin-1β (IL-1β)	Biolegend	579404	1 μg/ml, (Esquivel-Velazquez et al., 2015)
Interleucin-6 (IL-6)	Biolegend	570804	1 μg/ml, (Esquivel-Velazquez et al., 2015)
Interleucin-8 (IL-8, CXCL8)	Biolegend	574204	1 μg/ml, (Palacios-Arreola et al., 2014)
Laminin-111 (LAM1)	Sigma-Aldrich	I2020	80 / 100 μg/ml, (Insua-Rodriguez and Oskarsson, 2016)
Laminin-332 (LAM5)	Abcam	ab42326	20 μg/ml, (Insua-Rodriguez and Oskarsson, 2016)
Leptin	Sigma-Aldrich	L4146	1 μg/ml, (Garcia-Robles et al., 2013)
Lumican	Sigma-Aldrich	2846	1 μg/ml, (Nikitovic et al., 2014)
Melanoma growth stimulating activity alpha (GRO-α/CXCL1)	Sigma-Aldrich	G0657	1 μg/ml, (Palacios-Arreola et al., 2014)
Nidogen1	R&D systems	2570-nd	1 μg/ml, (Insua-Rodriguez and Oskarsson, 2016)
Osteopontin (OPN)	Novus Biologicals	NBC1-21056	1 μg/ml, (Insua-Rodriguez and Oskarsson, 2016)
Osteoprotegerin (OPG)	R&D systems	185-OS-025	1 μg/ml, (Weichhaus et al., 2015)
Stem cell factor (SCF)	R&D systems	255-SC-010	1 μg/ml, (Mimeault et al., 2007)
Stromal derived factor-1 (SDF-1β/CXCL12)	Abnova	P4470	1 μg/ml, (Palacios-Arreola et al., 2014)
Tenascin C (TNC)	Chemicon	CC065	1 μg/ml, (Insua-Rodriguez and Oskarsson, 2016)
Tumor growth factor β (TGFβ)	Biolegend	580704	1 μg/ml, (Esquivel-Velazquez et al., 2015)
Paclitaxel	Sigma-Aldrich	T7191	
rat tail Collagen type I	Ibidi	50201	
Collagen type 1, calf skin	Sigma-Aldrich	C8919	
Matrigel, growth factor reduced	Corning	356231	
**CRITICAL COMMERCIAL ASSAYS**			
RT^2^Profiler™ PCR arrays, human stem cell	Qiagen	PAHS-405ZF	
RT^2^Profiler™ PCR arrays, human epithelial to mesenchymal transition (EMT)	Qiagen	PAHS-090ZF	
Click-iT® Plus Edu imaging kit	Molecular probes	C10337	
RT^2^ SYBR Green PCR Master Mix	Qiagen	330503	
RT^2^-First Strand Kit	Qiagen	330401	
Quick-RNA MicroPrep	Zymo Research	R1050	
CellTiter-Glo 2.0 Assay	Promega	G9242	
**EXPERIMENTAL MODELS: CELL LINES**			
HMEC progression series	Dr. Martha Stampfer, Lawrence Berkeley national Laboratory, CA, USA	184	
**OLIGONUCLEOTIDES**			
RNAScope probe for *AXL*	Advanced cell diagnostics	Probe-Hs-AXL-C2	
RNAScope probe for *IL-8*	Advanced cell diagnostics	Probe-Hs-IL8-C1	
RNAScope probe for *OPN*	Advanced cell diagnostics	Probe-Hs-SPP1-C1	
**SOFTWARE AND ALGORITHMS**			
Cell profiler	www.cellprofiler.org		
R-language, R-studio	www.R-project.org/		
Cytobank	cellmass.cytobank.org		
IC50 toolkit	www.ic50.tk		

#### Immunohistochemistry

For *in vitro* immunofluorescence staining, cells were fixed in methanol:acetone (1:1) at −20°C for 20 min, blocked with PBS, 5% normal goat serum, 0.1% Triton X-100, and incubated with anti-Keratin14 (1:1,000, Covance, polyclonal) and anti-Keratin19 (1:200 AB7754, Abcam) overnight at 4°C, then visualized with fluorescent secondary antibodies (Invitrogen) incubated with sections for 2 h at room temperature.

Human formalin-fixed paraffin embedded (FFPE) tissue sections of normal mammary gland, invasive breast cancer and triple negative breast cancer (TNBC) were deparaffinized in xylene, and rehydrated according to standard protocols. Antigen retrieval was performed by boiling the sections in 0.01 M citrate buffer, pH6, for 25 min, followed by cooling to RT at the bench and a 10 min wash in dH_2_O prior to staining. For detection of ECM components, COL4 (1:100, MAB3326), COL1 (1:100, ab34710), LAM5 (1:50, MAB19562), Pan laminin (1:100, L9393), EPCAM (1:100, 34202), and K14 (1:1,000, Covance, polyclonal) antibodies were diluted in permwash buffer (BD Bioscience) and incubated at +4°C overnight. Fluorescence labeled secondary antibodies and Hoechst nuclei label were diluted also in permwash buffer and incubated 2 h at RT. For detection of COL6A3 (NBP-71566, Novus Biologicals) in FFPE tissue sections of normal mammary gland and triple negative breast cancer (TNBC) specimens, DAKO EnVision™ System-HRP (DAB) for Rabbit primary antibodies (K4011, DAKO) was applied according to the manufacturer's instructions. Antibodies were diluted in antibody-diluent with background reducing components (S3022, DAKO). Stained sections were counterstained with haematoxylin, prior to mounting using Faramount Aqueous Mounting Medium (S3225, DAKO). Images were obtained on a Leica DMLB microscope equipped with AnalySIS software (Leica).

#### Dual RNA *in situ* hybridization

Simultaneous *in situ* detection of the *OPN, IL-8* and *AXL* mRNA on human normal mammary FFPE tissue sections and TNBC specimens were performed using the RNA scope technology*. OPN* and *IL-8* were detected by C1 probes and *AXL* by C2-probes in all experiments. Probes and reagents were provided by Advanced Cell Diagnostics (ACD, Hayward, CA). Briefly, freshly cut 5-μm thick human archival mammary gland tissue sections were deparaffinized in xylene, followed by dehydration in an ethanol series. Tissue sections were then incubated in citrate buffer (0.01 M, pH 6) maintained at a boiling temperature (100–103°C) using a hot plate for 15 min, rinsed in deionized water, and immediately treated with10 μg/mL protease (Sigma-Aldrich, St. Louis, MO) at 40°C for 30 min in a HybEZ hybridization oven (Advanced Cell Diagnostics, Hayward, CA). Hybridization with target probes, preamplifier, amplifier, label probe and chromogenic detection were performed according to the ACD recommendations. Sections were counterstained with hematoxylin, and mounted with EcoMount prior to imaging. Assays using archival FFPE specimens were performed in parallel with positive and negative controls, to ensure interpretable and reproducible results.

#### Drug resistance assay

For paclitaxel resistance experiments, 8-well chamber slides were coated with COL1 (calf skin, Sigma-Aldrich) 100 μg/ml with or without COL6A3 (MyBioSource) 10 μg/ml diluted in 50 mM Hepes. 184AA3 cells were plated 24 h prior to drug treatment to coated chambers, followed by 24 h culturing with paclitaxel (0.1 μM, Sigma-Aldrich). Proliferation rate was analyzed by using Click-iT® Plus Edu imaging kit (Molecular probes). For paclitaxel IC50 analysis, 96-well plates were coated with COL1 (calf skin, Sigma-Aldrich) 100 μg/ml with or without OPN 4 μg/ml. 184AA3 cells were plated 4 h prior to drug treatment to coated wells and culture media was supplemented with or without IL-8 (50 ng/ml) and OPN (50 ng/ml). Followed by 48 h culturing with 5 different concentration of paclitaxel (0.001–1 μg/ml, Sigma-Aldrich). Cell viability was analyzed by using CellTiter-Glo 2.0 Assay (Promega). Paclitaxel was dissolved to DMSO, and control cultures were treated with equally diluted DMSO-solution.

#### Real-time PCR

Cells were cultured 24 h over the matrigel or COL1-gel (0.5 μg/ml, rat tail, non pepsinized, Ibidi) supplemented with or without 2 ug/ml COL6A3 (MyBioSource). Cell culture medium was supplemented with or without 10 ng/ml OPN (R&D systems), 10 ng/ml IL-8 (Abcam). Total RNA was purified with Trizol (Invitrogen). cDNA was synthesized with RT^2^ First strand kit (Qiagen). Transcripts levels were measured by RT^2^Profiler™ PCR arrays, human stem cell and human epithelial to mesenchymal transition (EMT) using RT^2^ SYBR Green PCR master mix (Qiagen) and LightCycler480 (Roche). Fold expressions were calculated using the formula 2^(−ΔΔCt)^, where ΔΔC_t_ is ΔC_t(sample)_−ΔC_t(control sample)_, ΔC_t_ is C_t(gene of interest)_−C_t(average from control gene setup)_ and C_t_ is the cycle at which the detection threshold is crossed.

#### Gene expression analysis

Total RNAs were isolated using Quick-RNA MicroPrep (Zymo Research). Sample preparation and Poly(A) enriched mRNA-sequencing were performed in City of Hope Comprehensive cancer center, Integrative genomics and bioinformatics core.

#### Data analysis

R was used for all statistical analysis (R foundation for statistical computing, Vienna, Austria. ISBN 3-900051-07-0, URL http://www.R-project.org/). To compare two population distributions *t*-tests were performed. Significance was established when: ^*^p < 0.05, ^**^p < 0.01, ^***^p < 0.001. RNA sequencing data is normalized and results are presented as RPKM (Reads Per Kilobase Million).

MEMA images were analyzed for single cell data with the CellProfiler (Carpenter et al., 2006) pipeline that is included in Supplementary File 1. Briefly fluorescence channel images were analyzed as separated gray scale images. To normalize intensity of images, threshold method: Background was used. This method finds the mode of the histogram part of the image, which is assumed to be the background of the image, and choose a threshold at twice that value. Threshold value was subtracted from the remaining pixel intensities. Marker-based watershed segmentation was used to identify single cells. Fluoresence intensity, cell size and morphology and cell neighbors were measured for each cell. Data analyses were performed using R-software. AXL and cKIT intensities were presented as mean of pixel intensity values. (AXL^+^/cKIT^+^)-subset was calculated by using threshold from COL4-microenvironment spots (mean+SD), cells expressing intensities above threshold were counted in (AXL^+^/cKIT^+^)-subset. Generalized linear model (GLM) was applied to decouple effects of multiple microenvironment components from each other, and then express (AXL^+^/cKIT^+^)-subset expression as a function of each microenvironment component. Tuckey's post Hoc test was performed after GLM to identify differences inside the microenvironmental factors. tSNE-method (Amir et al., 2013) in Cytobank portal (https://www.cytobank.org) was used to cluster and visualize MEMA data. For the clustering analysis, the mean value of each individual microenvironment was calculated from these data types: percentage of (AXL^+^/cKIT^+^)-subpopulation, fluorescein intensities in (AXL^+^/cKIT^+^)-subpopulation and in total population, cell number/spot, cell eccentricity and cell solidity.

IC50 was calculated by plotting and fitting data points to curve and regard the mid-point ligand concentration (IC50), curve fitting formula *y* = a+ [b-a]/[1+(x/c)^d^] is presented in ic50.tk.

## Additional resources

Additional information on 184 HMEC progression series: http://hmec.lbl.gov/mindex.html.

## Author contributions

TJ designed and performed the experiments and data analysis, also wrote the manuscript. AE performed COL6A3, *AXL, OPN*, and *IL-8* tissue immune staining's and RNAscope experiments and critically reviewed and helped with manuscript writing. AR provided technical support. FP provided support with data analysis and critically reviewed manuscript. JG provided support with HMEC culture system. MM performed RNA sequencing experiment. CT, DF, and LA provided breast tissue sections. MS provided support with HMEC culture system and critically reviewed manuscript. JL and ML supported, critically reviewed and helped with manuscript writing and experimental design.

### Conflict of interest statement

The authors declare that the research was conducted in the absence of any commercial or financial relationships that could be construed as a potential conflict of interest.

## Supplementary material

Supplementary File 1 Cell profile-pipeline used in this work.
